# Male Breast Cancer: The Three Decades' Experience of a Tertiary Care Hospital in a Lower-Middle Income Country

**DOI:** 10.7759/cureus.22670

**Published:** 2022-02-27

**Authors:** Sana Zeeshan, Tayyab Siddiqiui, Fatima Shaukat, Muhammad Usman Tariq, Nargis Khan, Lubna Vohra

**Affiliations:** 1 Surgery, Aga Khan University Hospital, Karachi, PAK; 2 Oncology, Aga Khan University Hospital, Karachi, PAK; 3 Histopathology, Prince Faisal Cancer Centre, King Fahd Specialist Hospital, Buraydah, SAU

**Keywords:** disease free survival, overall survival, clinicopathological features, male breast cancer, breast cancer

## Abstract

Introduction

Male breast cancer is uncommon and managed on the guidelines of female breast cancer due to tumor rarity. We sought to identify the incidence, clinicopathological features, and survival of all male breast cancer patients managed in our hospital.

Methods

A retrospective cross-sectional study was conducted at Aga Khan University Hospital (AKUH), Karachi, Pakistan, from January 1986 to December 2018. Demographic data, treatment records, and follow-up data of all male breast cancer patients who were treated at AKUH was reviewed.

Results

Thirty-eight out of 42 patients who presented over a period of 32 years were included. The mean age was 63 years. The most common tumor type and subtype were invasive ductal carcinoma (89.5%) and luminal A (73.7%), respectively. The majority (36.8%) of the patients presented at stage III. Among 30 (78.9%) patients who underwent surgery, mastectomy was performed in 30 (78.9%), upfront axillary clearance in 24 (63.2%), axillary sampling in five (15.1%) cases, and sentinel lymph node biopsy in one (2.6%) case. Neoadjuvant chemotherapy was given to 10 (26.3%) patients, and adjuvant chemotherapy to eight (21.1%) patients. Adjuvant hormonal treatment was administered to 22 (57.9%) patients, and 13 (34%) patients received adjuvant radiation to the chest wall. The five-year overall survival was 38.2% and the median survival was 36 months. The five-year disease-free survival (DFS) was found to be 33.7%.

Conclusion

Breast cancer in males presents at an advanced stage with poor survival. Multicenter studies are required to accurately identify incidence, prognostic factors, and outcomes in order to have a better understanding of its management.

## Introduction

Breast cancer is the most common cancer in women worldwide, with 2.4 million new cases diagnosed in 2016 [1]. Male breast cancer (MBC), on the other hand, is a rare disease with an incidence of 0.5-1% of all breast cancers, accounting for less than 1% of all malignancies in males [2,3]. Although it is an uncommon disease, its incidence is showing a rising trend similar to its female counterpart [3].

Most studies on MBC are retrospective cross-sectional studies with a small number of patients, and no randomized control trials have been conducted so far. Hence the approach to MBC is usually derived from female breast cancer studies [3]. Although breast carcinoma shares clinicopathological and prognostic features in both genders, there are some dissimilarities reported in the literature pertaining mainly, but not limited to, tumor biology, stage at presentation, and a decreasing trend after the age of 50 years [3-6]. MBC is known to be symptomatic at the time of presentation, usually advanced with a higher incidence of lymph-node involvement, hormone receptor positive in almost all cases, and with an absence of invasive lobular carcinoma type [3-6]. There is conflicting data on survival outcomes for male breast cancers. Some studies report worse outcomes for male breast cancer, while some show similar or even better survival when compared to female breast cancer [2,3,7,8]. Early diagnosis and advancement in treatment modalities have resulted in significantly improved survival in female breast cancer, but no such data exists for MBC [9,10].

Few studies on MBC presenting the epidemiology, tumor biology and outcomes have been published in Pakistan with the most recent publication in 2015. Hence, we aim to report our single institution’s updated data on MBC, which includes its frequency, clinicopathologic features, treatment, and survival.

This article was previously presented as a poster at the 20th Shaukat Khanum Cancer Symposium, held virtually on November 5-7, 2021.

## Materials and methods

A retrospective cross-sectional study was conducted which included all MBC patients diagnosed and treated at the Aga Khan University Hospital, Karachi, Pakistan, between January 1986 and December 2018. The inclusion criteria were the diagnosis of breast cancer in males from the histopathology section of our institute and treatment (tumor-related) from the department of surgery and/or department of oncology. Patients with a prior history of any other malignancy and patients with incomplete records were excluded from the study. The study was approved by the institution’s Ethics Review Committee (approval no. 2019-0391-2980). This study included 19 cases of MBC reported by Jamy et al. from the same study institution in 2009 [11]. Data on demographics, clinicopathological features, and treatment modalities of all MBC patients were retrieved from the institution’s breast cancer database. Follow-up information and survival data were obtained from the patients or patient’s attendant (in the case of the patient’s death) via telephonic conversation. All records were entered and analyzed using Statistical Package for the Social Sciences (SPSS) version-21 (IBM Corp., Armonk, NY).

The Shapiro-Wilk test was applied to check the hypothesis of normality for quantitative (continuous) variables. Quantitative (continuous) variables are expressed using descriptive statistics such as mean ± SD, median (interquartile range, IQR), skewness, maximum and minimum, and an appropriate unpaired t-test, or Mann-Whitney U test, was applied to check for a significant difference. Survival analysis was performed with Kaplan-Meier methods, while differences in survival between groups were assessed with log-rank tests. Multivariable analysis was performed using Cox regression. A P-value less than 0.05 was considered significant.

## Results

A total of 42 MBC patients were diagnosed and treated at our institution over the 32-year review period. We included and analyzed the data of 38 patients, as data was missing for four patients. The mean ± SD of age at presentation was 63 ± 12 years (range 40-86 years), with 21 (55.3%) patients more than 60 years of age. The most common presenting complaint was a palpable lump, which was noticed in 35 (92.1%) patients, followed by palpable ipsilateral axillary lymph nodes in 33 (86.8%) patients. Three (7.9%) patients presented with blood-stained nipple discharge. All patients had biopsy proven breast carcinoma in which 34 (89.5%) were invasive carcinoma, no special type (IDC, NST), while the remaining were single cases each of mucinous carcinoma, metaplastic squamous cell carcinoma, ductal carcinoma in situ (DCIS), and intracystic papillary carcinoma. T0 was comprised of two patients with nipple discharge; however, one had occult breast cancer with nodal disease while another had a few foci of DCIS. Twenty-one (55.3%) tumors were grade II and 17 (44.7%) tumors were grade III. The majority of the tumors were subtyped as luminal A (73.7%), followed by triple negative (18.4%) and human epidermal growth factor receptor (HER-2) type (7.9%). Based on the clinical prognostic stage as per American Joint Committee on Cancer (AJCC) 8th edition, the majority, i.e., 13 (34.2%) tumors were staged as IIIB, followed by stage IV in eight (21.1%) cases (Table 1).

**Table 1:** Clinicopathological characteristics of male breast cancer patients (n = 38). †Tx: where the size of the primary tumor couldn’t be determined due to prior incomplete excision of the tumor at an outside institution. *IDC/ICNST: invasive ductal carcinoma, now known as invasive mammary carcinoma of no special type.

Characteristics	Expression
Patient’s age	
Range	40-86 years
Mean age ± SD	63 ± 12 years
Presenting complaint	
Lump	35 (92%)
Nipple discharge	3 (8%)
T stage at presentation	
T0	2 (5.3%)
T1	2 (5.3%)
T2	19 (50%)
T3	3 (7.9%)
T4	11 (28.9%)
Tx^†^	1 (2.6%)
Clinical axillary nodal status at presentation	
Lymph nodes involved	33 (86.8%)
Lymph nodes not involved	5 (13.2%)
Tumor stage	
0	2 (5.3%)
Ia	2 (5.3%)
Ib	3 (7.9%)
IIa	4 (10.5%)
IIb	5 (13.2%)
IIIa	1 (2.6%)
IIIb	13 (34.2%)
IV	8 (21.1%)
Histological tumor grade	
II	21 (55.3%)
III	17 (44.7%)
Histological tumor type	
IDC/ICNST*	34 (89.5%)
Others	4 (10.5%)
Molecular sub-type	
Luminal A	28 (73.7%)
HER type	3 (7.9%)
Triple negative	7 (18.4%)

Surgery was performed on all 30 non-stage IV patients (78.9%), and all of them underwent a mastectomy while the remaining eight (21.1%) patients did not undergo any surgical intervention because of metastatic disease. A total of 33 patients, including stage IV, had lymph node involvement at presentation. Twenty-four (63.2%) of the 30 non-stage IV patients underwent upfront axillary clearance, five (15.1%) underwent sampling at the primary surgeon’s discretion, and one (2.6%) patient underwent sentinel lymph node biopsy as it was stage 1, clinically N0 disease (Table 2).

Six out of eight stage IV patients received primary systemic chemotherapy, while one received primary hormonal treatment. One of the stage IV patients passed away before the initiation of any treatment. In the non-stage IV patients, neoadjuvant chemotherapy was administered to 10 (26.3%) patients and adjuvant chemotherapy to eight (21.1%) patients, whereas 14 patients did not receive chemotherapy either because of early stage, presence of only DCIS, old age, or not medically fit to tolerate chemotherapy. The chemotherapy regimens were comprised mostly of Adriamycin, Cyclophosphamide, and Taxol; 5-fluorouracil, doxorubicin, and cyclophosphamide (FAC); cyclophosphamide, methotrexate, fluorouracil (CMF), and taxotere and cyclophosphamide (TC), similar to the female breast cancer chemotherapy protocol. Adjuvant hormonal treatment was received by 22 (57.9%) patients, and 13 (34%) patients received adjuvant radiation to the chest wall with a mean dose of 60 Gy in 30 fractions (Table 2). Five of the luminal A tumor patients did not receive any hormonal treatment, as two patients declined further treatment, two were lost to follow-up, and one passed away before the initiation of treatment.

**Table 2:** Summary of treatment information of male breast cancer patients (n = 38).

Treatment	Frequency (%)
Surgery	
Mastectomy	30 (78.9%)
Axillary clearance	24 (63.2%)
Axillary sampling	5 (15.1%)
Sentinel lymph node biopsy	1 (2.6%)
Chemotherapy	24 (63.2%)
Neoadjuvant chemotherapy	10 (26.3%)
Adjuvant chemotherapy	8 (21.1%)
Primary chemotherapy	6 (15.8)
Radiotherapy	13 (34.2%)
Hormonal therapy	23 (60.5%)
Adjuvant hormonal therapy	22 (57.9%)
Primary hormonal therapy	1 (2.6%)

**Figure 1:**
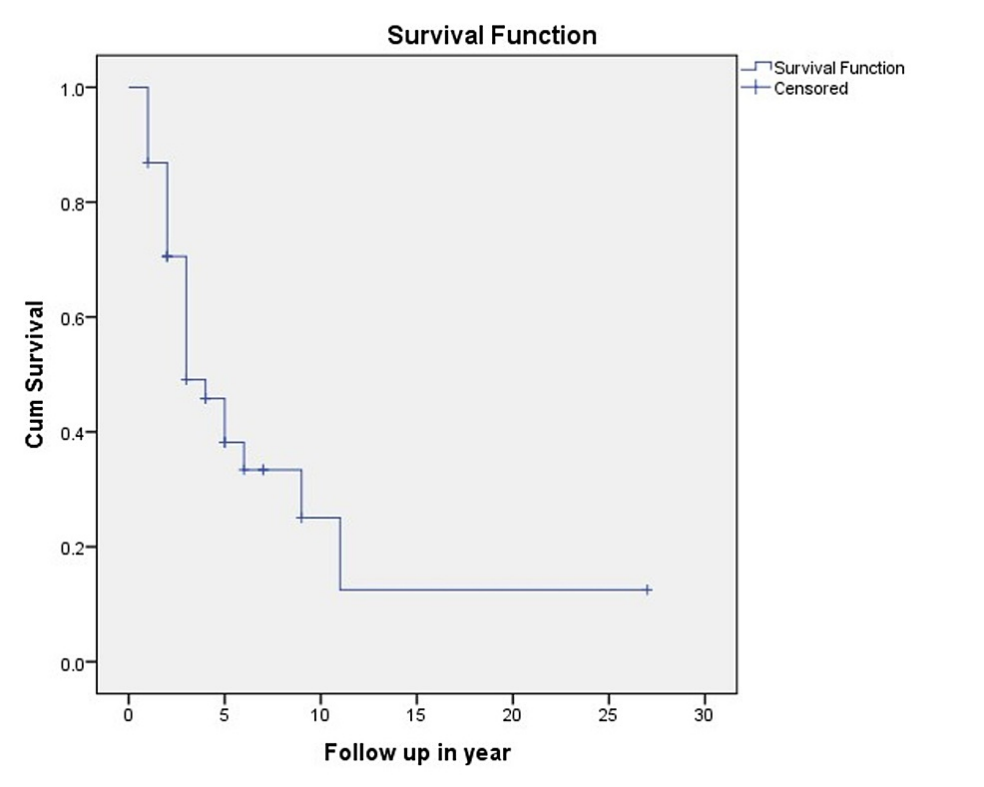
Kaplan-Meier curve demonstrating overall survival in male breast cancer.

Follow-up was completed for all patients via telephonic calls. The five-year overall survival (OS) was 38.2% and the median survival was 36 months. Overall survival (OS) varied by stage. Patients with stage I disease had a five-year OS of 100% compared to 59.4% with stage II, 22.8% with stage III, and 12.5% with stage IV. The five-year disease-free survival (DFS) was found to be 33.7%. After excluding stage IV disease, 17 out of 30 patients (44.73%) had a recurrence, out of which two were local recurrences on the chest wall and 15 were distant recurrences. Out of these 17 recurrences, 15 occurred within the first three years of diagnosis, seven, six and two within the first, second and third years, respectively. Patients with DCIS and stage I had no recurrence. In stage II, six patients had recurrent disease, out of which two were local recurrences and four were distant recurrences. In stage III, all 11 patients had distant recurrences. Fifteen patients with distant recurrences showed bone involvement in five (33.3%), both lung and liver involvement in two (13.3%), only lung involvement in five (33.3%), and brain in three (20%) patients. The overall survival of MBC is demonstrated in Figure 1.

## Discussion

As documented in different studies [12-14], MBC is a rare disease with a worldwide reported incidence of less than 1%. Khokher et al. from their hospital-based breast cancer registry of 6,718 patients at one of the oncology centers in Pakistan have reported a 2% incidence of MBC over 10 years (2000-2009) [15]. Some African countries like Tanzania and Zambia have reported even higher rates of 6% to 15% [16,17]. We observed the incidence of 0.8% in our study. Since Pakistan lacks a national cancer registry, it is difficult to state the exact population-based incidence and pattern of local disease in MBC and only institution-based data has been published so far, making true comparisons with developed countries challenging.

The risk of MBC increases with age and is usually diagnosed in an older population as compared to female breast cancer [2,14]. Most international studies have reported the median age of MBC as 67-69 years, while the median age for the diagnosis of female breast cancer is reported as 57-61 [18]. In contrast, a retrospective review of 19 MBC cases diagnosed between 2000 and 2009 by Jamy et al. reported a mean age of 52 years [11]. Sharif et al. also reported an average age of 57 years in their study of 55 patients from Northern Pakistan [19]. Our results were closer to the international data, with a mean age of 63 years. The difference in age at diagnosis between males and females is mainly due to the implementation of screening mammography in women, which results in the early detection of breast cancer in female patients [14].

The majority of our patients presented with palpable, painless lumps as initial symptoms, which is also mentioned in other studies [4,10,11]. Other presenting complaints included nipple discharge, ulceration, retraction, and bleeding [11]. Differential diagnosis of MBC includes, gynecomastia, pseudo-gynecomastia and rarely sarcoma [20]. Due to these differentials and low level of suspicion, MBC is usually diagnosed at an advanced stage with axillary lymph node involvement at presentation.

The most common breast cancer in males is invasive breast carcinoma, NST as observed in our study too. They found out that only four patients had mucinous carcinoma, metaplastic squamous cell carcinoma, intracystic papillary carcinoma, and ductal carcinoma in situ (DCIS). Of note, invasive lobular carcinoma (ILC) in men is an extremely rare entity, which has been reported only in a few case reports, and it is commonly associated with Klinefelter syndrome [21]. Only one study from Pakistan has reported two cases of ILC out of their 55 cases of MBC [19]. None of the cases in our cohort were diagnosed as ILC. Furthermore, none of the tumors in our study were grade 1, and none was luminal B or triple positive subtype.

Standard surgical management of MBC over the past few years has been modified radical mastectomy (MRM). Neoadjuvant chemotherapy is now offered to patients with locally advanced disease in order to downstage the tumor [4,22]. The role of breast conserving surgery (BCS) is limited in MBC as compared to female breast cancer. BCS in the male breast is opted only in early stage cancers where adequate resection of margins can be achieved, which is practically difficult to attain due to the small size of the breasts in males [22]. Acceptance of sentinel node biopsy after down staging of clinically positive axilla, similar to management in female breast cancer, is now being considered in MBC as well [23].

MRM was the most frequently performed surgical procedure in our study. Due to a scarcity of data, the role of adjuvant treatment with either chemotherapy, hormonal therapy, or radiotherapy has not been evaluated in randomized trials. There are no established guidelines for the role of adjuvant treatment in MBC. Lack of international guidelines on adjuvant management of MBC results in poor DFS in terms of distant and local failure [24].

Over the last few years, the survival of female breast cancer has improved significantly. This is likely a combined result of earlier detection with better imaging modalities, screening programs and improvements in treatment of female breast cancer [2]. Contrary to this, MBC has lower survival rates due to higher age at diagnosis, locally advanced disease at presentation, and limited established literature for its management [14,24].

We acknowledge that our study has limitations due to its single-centered retrospective design, which limited our ability to assess overall incidence in the Pakistani male population and survival on all parameters. Our study included 19 cases already reported by Jamy et al. in 2015 [11], and we report extended follow-up of these patients. Exact details regarding systemic therapy in terms of dosage, duration, and compliance could also not be ascertained, which have a significant effect on disease outcomes. Strengths of our study include the long follow-up time, high-quality reporting of tumors by dedicated breast pathologists, and patient management as per international standards.

## Conclusions

Even though breast cancer occurs uncommonly in men, its management remains complex and unclear. Male breast cancer presentation is different than females and due to lack of data, they are managed on the lines of female breast cancer. Therefore, further research will be necessary, both at the clinical as well as molecular level, to establish the most appropriate therapies. This will prove challenging, especially in lower-income countries where breast cancer still remains a social taboo, resulting in its presentation at an advanced stage. Since males do not qualify for breast cancer screening due to the rarity of the disease, awareness regarding the presentation and disease occurrence is of utmost importance to reduce the incidence of late presentation and mortality associated with it.
